# Prevalence of Shiga Toxin-Producing *Escherichia coli* O157 and Non-O157 Serogroups Isolated from Fresh Raw Beef Meat Samples in an Industrial Slaughterhouse

**DOI:** 10.1155/2021/1978952

**Published:** 2021-12-15

**Authors:** Kiandokht Babolhavaeji, Leili Shokoohizadeh, Morteza Yavari, Abbas Moradi, Mohammad Yousef Alikhani

**Affiliations:** ^1^Department of Microbiology, Faculty of Medicine, Hamadan University of Medical Sciences, Hamadan, Iran; ^2^Department of Clinical Sciences, Faculty of Veterinary Sciences, Bu-Ali Sina University, Hamadan, Iran; ^3^Department of Community Medicine, School of Medicine, Hamadan University of Medical Sciences, Hamadan, Iran

## Abstract

**Background:**

The aims of the current study are the identification of O157 and non-O157 Shiga Toxin-Producing *Escherichia coli* (STEC) serogroups isolated from fresh raw beef meat samples in an industrial slaughterhouse, determination of antimicrobial resistance patterns, and genetic linkage of STEC isolates.

**Materials and Methods:**

A total of 110 beef samples were collected from the depth of the rump of cattle slaughtered at Hamadan industrial slaughterhouse. After detection of *E. coli* isolates, STEC strains were identified according to PCR for *stx1*, *stx2, eaeA*, and *hlyA* virulence genes, and STEC serogroups (O157 and non-O157) were identified by PCR. The genetic linkage of STEC isolates was analyzed by the ERIC- (Enterobacterial Repetitive Intergenic Consensus-) PCR method. The antimicrobial susceptibility of STEC isolates was detected by the disk diffusion method according to CLSI guidelines.

**Results:**

Among 110 collected beef samples, 77 (70%) were positive for *E. coli*. The prevalence of STEC in *E. coli* isolates was 8 (10.4%). The overall prevalence of O157 and non-O157 STEC isolates was 12.5% (one isolate) and 87.5% (7 isolates), respectively. The hemolysin gene was detected in 25% (2 isolates) of STEC strains. Evaluation of antibiotic resistance indicated that 100% of STEC isolates were resistant to ampicillin, ampicillin-sulbactam, amoxicillin-clavulanic acid, and cefazolin. Resistance to tetracycline and ciprofloxacin was detected in 62.5% and 12.5% of isolates, respectively. The analysis of the ERIC-PCR results showed five different ERIC types among the STEC isolates.

**Conclusion:**

The isolation of different clones STECs from beef and the presence of antibiotic-resistant isolates indicate that more attention should be paid to the hygiene of slaughterhouses.

## 1. Introduction

Shiga toxin-producing *Escherichia coli* (STEC) is an important cause of gastrointestinal disease in humans. Infections may result in life-threatening disease, e.g., hemolytic-uremic syndrome (HUS) and thrombotic thrombocytopenic purpura (TTP), due to the consumption of undercooked ground beef and other beef products contaminated with STEC [1]. Although several routes exist for human infection with STEC, beef remains the main source. Because of the nature of the food supply, safety concerns with beef will continue, and the challenges faced by the beef industry will increase at the production and processing levels [2]. Emerging evidence indicates that the *E. coli* Shiga toxins (stx) constitute a family of several related cytotoxins. At least two of them, Stx1 and Stx2, which are encoded by *stx1* and *stx2* genes, respectively, are known to be associated with human disease [3]. *E. coli* O26, O45, O91, O103, O111, O113, O121, O128, O145, and O157 are the major STEC serogroups, and all are able to produce Shiga toxins. Some isolates also have *eaeA* and/or *hlyA* in addition to *stx1* and *stx2*. The *eaeA* gene is coded to a protein called intimin, the function of which is to create attaching/effacing lesions (A/E) [4]. In addition to the expression of Shiga toxins and the locus of enterocyte effacement (LEE) pathogenicity island, *hlyA* is a commonly used marker for the detection of potentially pathogenic E. coli strains, although its exact role in pathogenesis is not completely understood [5]. In Iran, most studies have focused on STEC in dairy and animal stool samples, and little information is available on the prevalence of STEC in fresh beef. No prior research on STEC prevalence has been carried out at the industrial slaughterhouse in Hamadan, western Iran. Thus, this study is conducted to answer questions about the frequency of STEC O157 and non-O157, *stx1*, *stx2*, *eaeA,* and *hlyA* virulence genes and to evaluate the antibiotic resistance and genetic linkage of the STEC isolated from fresh meat beef of cattle slaughtered at Hamadan industrial slaughterhouse.

## 2. Materials and Methods

### 2.1. Sampling and Identification of *Escherichia coli* Strains

This cross-sectional study was conducted at Hamadan Industrial Slaughterhouse from February to June 2020. This study was approved by the Ethics Committee of Hamadan University of Medical Sciences (IR.UMSHA. REC.1398.1020). Samples were collected from the available slaughtered cattle at the time of sampling. Beef samples were collected from the rump, cut to approximate dimension 10 cm × 10 cm × 3 cm (∼10 grams of sample), and obtained in each case by opening cuts through beef of the slaughtered cattle. The knife was disinfected by alcohol prior and subsequent to taking each sample. The samples were placed into sterile plastic, preserved in a cold box containing ice, and prepared for transport to the microbiology research laboratory at the Department of Microbiology, Hamadan University of Medical Sciences. 2 ml of sterile Phosphate Buffered Saline (PBS) was added to each sample prior to removal from the plastic container, and the beef samples were homogenized, poured into Falcon 50 ml tubes containing 10 ml sterile Nutrient Broth (NB) media, and incubated overnight. The samples were cultured on Eosin Methylene Blue (EMB) and Sorbitol MacConkey agar and incubated at 37°C for 24 hours. Gram-negative bacilli phenotypically were identified as STEC using an array of biochemical tests such as IMVIC and sorbitol fermentation assays [6].

### 2.2. Molecular Detection of Virulence Genes

A sweep of three *E. coli* colonies on EMB agar were inoculated in Tripticase Soy Broth (TSB) and incubated overnight at 37°C. The genomic DNAs of the *E. coli* colonies were extracted by boiling [7]. Virulence genes including *stx1*, *stx2*, *eaeA, hlyA*, and STEC serogroups O157, O145, O128, O121, O113, O111, O103, O91, O45, and O26 encoding genes were detected by PCR using specific primers as described previously [8] in a reaction mixture with a total volume of 25 *μ*L in a thermocycler (Bio-Rad, Inc., USA). Multiplex PCR was conducted to detect *stx1*, *stx2*, and *eaeA* genes. The *hlyA* gene was identified distinctively. Enteropathogenic *E. coli* or EPEC strains are positive for *eae* and negative for *stx*1 and *stx*2, and STEC strains are *stx*1 and/or *stx*2 and eae+/eae. The PCR reactions for *stx1, stx2*, and *eaeA* genes consisted of an initial denaturation step at 94°C for 10 minutes (*hly*: 94°C, 10 min), followed by 30 cycles of 60 sec at 94°C (*hly*: 94°C, 1 min), annealing for 60 sec at 55°C (*hly:* 60°C, 2 min), and extension for 60 sec at 72°C (*hly*: 72°C, 2 min). A final extension step was performed at 72°C for 7 min (*hly*: 72°C for 5 min). The PCR reactions for serogroups were prepared as described previously [9]. The PCR products were separated by electrophoresis in 1.2% agarose gels. The results of PCR were confirmed by PCR product sequencing. The PCR products were sequenced by a company (Macrogen/Korea) and aligned in the Basic Local Alignment Search Tool (BLAST) in the NCBI.

### 2.3. Antimicrobial Susceptibility Testing

The antimicrobial susceptibility of STEC isolates to ampicillin (AMP), amoxicillin-clavulanic acid (AMC), tetracycline (TE), cefazolin (CZ), trimethoprim-sulfamethoxazole (SXT), ampicillin-sulbactam (SAM), gentamicin (GEN), imipenem (IPM), ceftriaxone (CRO), ciprofloxacin (CIP), and ceftazidime (CAZ) disks (MAST Group, UK) was detected by the disk diffusion method according to CLSI 2018 guidelines [10].

### 2.4. ERIC-PCR

Genetic relatedness of STEC isolates were investigated by the ERIC-PCR technique. This technique was carried out in a thermocycler (Bio-Rad, Inc. USA) using the primer ERIC (F): 5ʹ-ATG TAA GCT CCT GGG GAT TCAC-3ʹ and ERIC (R): 5ʹ-AAG TAA GTG ACT GGG GTG AGC G3ʹ (Metabion, Germany) as described previously [11]. The ERIC patterns of bands on agarose gel were analyzed by online data analysis service (inslico.ehu.es). ERIC patterns were clustered by the UPGMA program and compared using the Dice method [11].

## 3. Results

### 3.1. Characterization of STEC Isolates

**Figure 1:**
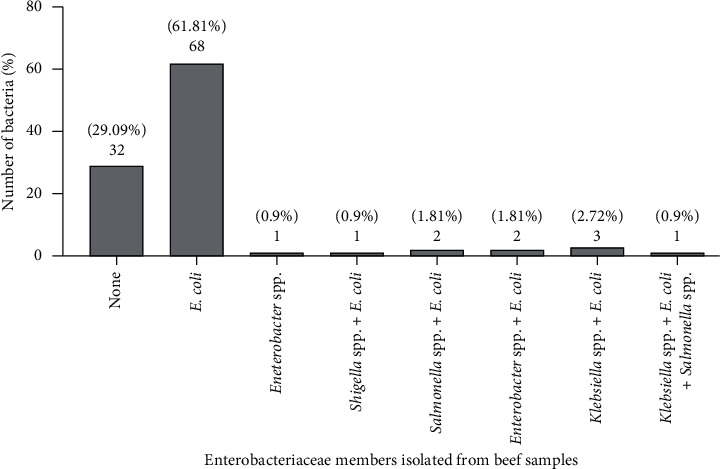
The prevalence of Enterobacteriaceae members isolated from beef samples. 1- *E. coli*; 2- *Shigella* spp. + *E. coli*; 3- *Salmonella* spp. + *E. coli*; 4- *Enterobacter* spp.; 5- *Salmonella* spp. + *Klebsiella* spp. + *E. coli*; 6- culture negative; 7- *Klebsiella* spp. + *E. coli*; and 8- *Enterobacter* spp. + *E. coli*.

Among the 110 beef samples, 77 isolates (70%) were identified as *E. coli*. Culture-negative plates were detected in 32 samples (29.1%), and pure *E. coli* was detected in 68 samples (61.8%). In addition to *E. coli*, other Enterobacteriaceae members were isolated from mixed cultures of beef samples (Figure 1).

**Figure 2:**
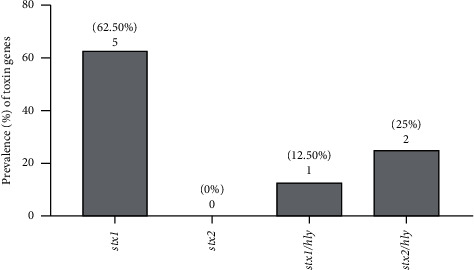
The prevalence of virulence gene patterns detected in 8 STEC isolates.

Based on molecular and microbiological tests, 8 (10.4%) and one (1.2%) of the *E. coli* isolates were characterized as STEC and EPEC, respectively. The *stx*1, *stx*1/*hly*, and *stx*2*/hly* were detected in 5 (62.5%), 1 (12.5%), and 2 (25%) of the STEC isolates, respectively. The *hly*A and *stx*2 genes were detected in 2 (25%) STEC isolates. The prevalence of virulence genes patterns in 8 STEC isolates is illustrated in Figure 2.

On the basis of the molecular detection of STEC serogroups, one STEC isolate belonged to the O157 serogroup and 7 isolates were categorized as non-O157 serogroups. Based on the PCR results, one EPEC strain was detected by PCR which was positive for the *eae*A gene. Analysis of sequencing results indicated that the *stx2* gene was 99% identical to the standard strain, while the *wzx* and *stx1* genes were 97% and 96% identical to their standard strains, respectively.

### 3.2. Antimicrobial Sensitivity Patterns

The results of the antimicrobial susceptibility test conducted on 8 STEC isolates found that all were resistant to ampicillin, ampicillin-sulbactam, amoxicillin-clavulanic acid, and cefazolin. Resistance to tetracycline and ciprofloxacin was detected in 5 and 2 of isolates, respectively. All 8 of STEC isolates were susceptible to gentamicin, imipenem, trimethoprim-sulfamethoxazole, ceftriaxone, and ceftazidime.

### 3.3. STEC ERIC-PCR Typing

**Figure 3:**
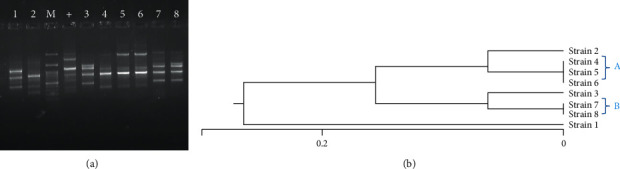
(a) ERIC-PCR patterns of STEC isolates on gel electrophoresis. Lane 1 and 2: STEC isolates, lane 3: DNA size marker, lane 4: positive control, and lanes 3–8: STEC isolates. (b) Dendrogram of analysis of ERIC results.

Analysis of ERIC-PCR results showed ≥80–100% similarity among STEC isolates (Figure 3). Genetic diversity was established among 8 STEC isolates by detecting 5 different ERIC profiles with a similarity cutoff of ≥95%. In total, we identified 5 different ERIC profiles, including two common types (A and B) and 3 unique types. All 5 isolates in common types A and B harbored the *stx1* gene. Unique types of STEC isolates showed different toxin profiles, i.e., *hly*/*stx*1 (one isolate) and *hly*/*stx*2 (two isolates). The isolate with serogroup O157 showed the unique or single ERIC type. The profiles of ERIC, toxins, and antibiotics of 8 different STEC isolates are compared in Table 1.

## 4. Discussion

Cattle are considered the major reservoir of Shiga toxin-producing *E. coli.* This research is the first study in which STEC isolates were isolated and characterized in fresh beef samples from the industrial slaughterhouse in Hamadan, western Iran. In our study, the overall STEC prevalence was 10.4% of the samples, only one STEC isolate was in the O157 serogroup, and 87.5% were non-O157 strains. Our results are within the range of prevalence of STEC in beef samples of prior studies. Osaili et al. [12] found that 7.8% of beef samples were polluted with O157: H7 STEC in Jordan, while much lower positive incidence was found in raw meat samples from Egypt (3.4%) and in Saudi Arabia (2%) [13, 14]. Our results indicate an occurrence of the serogroup O157 (1.3%) which is comparable to 1.35% reported by Yousefi et al. from Mashhad in Iran [15] and lower than a study conducted in Isfahan, Iran, which reported 6.4% *E. coli* O157 in bovine carcass samples [16]. Elsewhere in Iran, Momtaz et al. reported 238 (29.02%) ruminant meat samples were positive for the presence of *E. coli*. They evaluated O157 and non-O157 serogroups of STEC, included O26, O103, O111, O145, O45, O91, O113, O121, and O128 in beef samples, and reported the presence of all these serogroups in their results [9].

The prevalence of the serogroup O157 in our study was slightly higher than in studies conducted in other countries, i.e., 0.3% in the European Union [17], 0.8% in the United States [18], and 1% in Brazil [19], and lower than the prevalence of 2.2% in Nigeria [20] and 1.7% in Australia [21]. Samadpour et al. detected STEC in 23% of beef samples, and the serogroup O157 was not detected in any of the beef samples [22]. Differences in the prevalence of STEC in these studies likely result from differences in research protocols, including sampling methods (e.g., site on beef and number/surface sites sampled) and animal history (e.g., origin, cleanliness, season, and age). Based on the seasonal occurrence of bacteria, literature data indicate that the prevalence of *E. coli* O157 shedding in cattle appears to be more common in the warmer months [23–25]. In our study, the isolation of *E. coli* as well as STEC strains was higher in the spring. The age of cattle is one of the items which may influence the prevalence of STEC, as Renter et al. found the prevalence of STEC O157 in feeder cattle is higher than in cow calf or dairy cattle [26]. In our study, most cattle were feeder cattle with near-uniform age.

Resistance to antibiotics is a major concern for animal and human health. Antibiotic-resistant *E. coli* can spread from food-producing animals to humans, and for this reason, we explored the antimicrobial susceptibility of STEC isolates. In this study, we detected full resistance to ampicillin, ampicillin-sulbactam, amoxicillin-clavulanic acid, and cefazolin and also resistance to tetracycline and ciprofloxacin in 5 and 2 of these isolates, respectively. Based on these findings, some beta-lactam antibiotics and tetracycline should not be considered as the first therapeutic choice for treatment of gastrointestinal infections due to *E. coli*. In contrast, gentamicin, imipenem, trimethoprim-sulfamethoxazole, ceftriaxone, and ceftazidime were found to be most effective on STEC isolates. Previous studies also reported the rates of resistance to antibiotics in STEC strains. In a study from Egypt, approximately half of the STEC isolates were multidrug resistant. The highest antimicrobial resistance rates were found against nalidixic acid (51.4%) and ampicillin (48.6%), whereas the lowest rates were reported against gentamicin (5.7%) and ciprofloxacin (11.4%) [27]. Momtaz et al. reported high-level resistance to penicillin and tetracycline and low-level resistant to ciprofloxacin in STEC isolated from raw beef samples collected from ruminants including beef, sheep, goat, and camel [9].

In a study from Iran, Ranjbar et al. found that 100% of STEC isolated from hospital foods were resistant to ampicillin (similar to our results) and 100% of the isolates were resistant to tetracycline (our study found 62.5% of isolates were resistant). Ranjbar et al. also declared that half of the STEC isolates were resistant to ciprofloxacin and trimethoprim-sulfamethoxazole [7], whereas in our study, 100% of isolates were susceptible to trimethoprim-sulfamethoxazole and only 12.5% of them were resistant to ciprofloxacin.

In the current study, ERIC-PCR genotyping demonstrated 5 different ERIC-genotypes from 8 STEC isolates. Therefore, the results of ERIC-PCR typing showed genetic diversity among STEC isolates as well as the different potential sources of contamination with STEC strains. According to ERIC-PCR results, the common types showed similar toxin profiles and close antibiotic-resistant patterns and serogroups (non O157). One of the unique or single ERIC type showed a different serogroup (O157), toxin profile, and antibiotic pattern from other isolates. In the other studies, diversity in STEC isolates was established. Noureldaim et al. found that STEC strains isolated from frozen beef samples sold in Malaysian hyper- and supermarkets had originated in India and Australia. STEC strain was categorized into 4 clusters and 2 single isolates at a similarity level of 80%, and based on the results in their study, ERIC-PCR discriminated the isolates better than the Random Amplified Polymorphic DNA-PCR (RAPD) method [28].

Elmonir et al. used a dendrogram map to classify the clinical STEC isolates (from cattle and humans) and food products (milk and beef) from Egypt into 27 different ERIC genotypes. The isolates that belonged to the same serotype were clustered together [27].

There were some limitations in this study due to the need to compare to human samples and the lack of positive control for non-O157 serogroups.

One of the most important limitations of the present study was the small number of fresh beef samples because the COVID-19 pandemic in Iran limited both the sampling process and financial support. Although the ERIC-PCR technique was able to isolate STEC isolates, there are more powerful techniques such as PFGE and MLST. Due to the high cost in terms of equipment and analysis, we selected a cheaper, simpler, and more accessible technique. For future studies and to obtain more accurate and reliable results, the use of more powerful techniques such as MLST and PFGE is suggested.

## 5. Conclusions

The results of the current study revealed that fresh beef in slaughterhouses can be reservoirs of Shiga toxin-producing *E. coli*. The results of the current and prior research indicate the usefulness of the ERIC-PCR method in the investigations of genetic linkages of STEC isolates. The isolation of STEC from fresh beef and the presence of antibiotic-resistant isolates lead us to recommend that greater attention be paid to the hygiene of slaughterhouses, in order to control any unsanitary methods in slaughterhouses, and cooked meat products. We further recommend longitudinal studies of slaughterhouse hygiene in order to monitor key trends in biotic communities.

## Figures and Tables

**Table 1:** Comparison of toxins, antibiotic resistance, ERIC profiles, and serogroups of STEC isolates.

STEC isolates	ERIC types	Antibiotic-resistance patterns	Toxin profiles	Serogroup
1	Single	AMP/SAM/AMC/CZ/TE/SXT/CIP	*hlyA/stx1*	Non-O157
2	Single	AMP/SAM/AMC/CZ/TE/CIP	*hly/stx2*	O157
3	Single	AMP/SAM/AMC/CZ/TE	*hly/stx2*	Non-O157
4	A	AMP/SAM/AMC/CZ/TE	*stx1*	Non-O157
5	A	AMP/SAM/AMC/CZ/TE	*stx1*	Non-O157
6	A	AMP/SAM/AMC/CZ	*stx1*	Non-O157
7	B	AMP/SAM/AMC/CZ	*stx1*	Non-O157
8	B	AMP/SAM/AMC/CZ/TE	*stx1*	Non-O157

AMP: ampicillin; SAM: ampicillin-sulbactam; AMC: amoxicillin- clavulanic acid; CZ: cefazolin; TE: tetracycline; SXT: trimethoprim-sulfamethoxazole; CIP: ciprofloxacin.

## Data Availability

The data used to support the findings of this study are included within the article.

## Abbreviations

STEC: Shiga toxin-producing *Escherichia coli*
*E.coli*: *Escherichia coli*
HUS: Hemolytic-uremic syndrome
TTP: Thrombocytopenic purpura
LEE: Locus of enterocyte effacement
A/E: Attaching/effacing lesions
NB: Nutrient broth
EMB: Eosin methylene blue
ERIC: Enterobacterial repetitive intergenic consensus
EPEC: Enteropathogenic *E. coli*.

## Supplementary Materials

Supplementary Materials See the supplementary materials for the sequencing results of PCR products.

## References

[1] Paton A. W., Paton J. C. (1998). Detection and characterization of shiga toxigenic Escherichia coli by using multiplex PCR assays for stx1, stx 2, eaeA, enterohemorrhagic E. coli hlyA, rfb O111, and rfb O157. *Journal of Clinical Microbiology*.

[2] Hussein H. S. (2007). Prevalence and pathogenicity of Shiga toxin-producing *Escherichia coli* in beef cattle and their products. *Journal of Animal Science*.

[3] Karmali M. A. (1989). Infection by verocytotoxin-producing *Escherichia coli*. *Clinical Microbiology Reviews*.

[4] Paton J. C., Paton A. W. (1998). Pathogenesis and diagnosis of Shiga toxin-producing *Escherichia coli* infections. *Clinical Microbiology Reviews*.

[5] Schwidder M., Heinisch L., Schmidt H. (2019). Genetics, toxicity, and distribution of enterohemorrhagic *Escherichia coli* hemolysin. *Toxins*.

[6] Mahon C. R., Lehman D. C., Manuselis G. (2018). *Textbook of Diagnostic Microbiology-E-Book*.

[7] Ranjbar R., Masoudimanesh M., Dehkordi F. S., Jonaidi-Jafari N., Rahimi E. (2017). Shiga (Vero)-toxin producing *Escherichia coli* isolated from the hospital foods; virulence factors, o-serogroups and antimicrobial resistance properties. *Antimicrobial Resistance and Infection Control*.

[8] Monday S. R., Beisaw A., Feng P. C. H. (2007). Identification of Shiga toxigenic *Escherichia coli* seropathotypes A and B by multiplex PCR. *Molecular and Cellular Probes*.

[9] Momtaz H., Safarpoor Dehkordi F., Rahimi E., Ezadi H., Arab R. (2013). Incidence of Shiga toxin-producing *Escherichia coli* serogroups in ruminant’s meat. *Meat Science*.

[10] Clinical and Laboratory Standards Institute (2018). Performance standards for antimicrobial susceptibility testing. *CLSI Supplement M100*.

[11] Zarei O., Shokoohizadeh L., Hossainpour H., Alikhani M. Y. (2018). Molecular analysis of *Pseudomonas aeruginosa* isolated from clinical, environmental and cockroach sources by ERIC-PCR. *BMC Research Notes*.

[12] Osaili T. M., Alaboudi A. R., Rahahlah M. (2013). Prevalence and antimicrobial susceptibility of Escherichia coli O157:H7 on beef cattle slaughtered in Amman abattoir. *Meat Science*.

[13] Ahmed A. M., Shimamoto T. (2014). Isolation and molecular characterization of Salmonella enterica, Escherichia coli O157:H7 and Shigella spp. from meat and dairy products in Egypt. *International Journal of Food Microbiology*.

[14] Hessain A. M., Al-Arfaj A. A., Zakri A. M. (2015). Molecular characterization of Escherichia coli O157:H7 recovered from meat and meat products relevant to human health in Riyadh, Saudi Arabia. *Saudi Journal of Biological Sciences*.

[15] Yousefi E., Ghouchannezhad Nournia B., Yousefi A., Fakour F. (2019). Identification of *Escherichia coli* O157: H7 from slaughtered beef in Mashhad using biochemical and molecular methods. *Iranian Journal of Medical Microbiology*.

[16] Rahimi E., Momtaz H., Hemmatzadeh F. (2008). The prevalence of *Escherichia coli* O157:H7, Listeria monocytogenes and Campylobacter spp. on bovine carcasses in Isfahan, Iran. *IJVR*.

[17] EFSA (European Food Safety Authority) (2013). The European Union summary report on trends and sources of zoonoses, zoonotic agents and food-borne outbreaks in 2011. *EFSA Journal*.

[18] Hill W. E., Suhalim R., Richter H. C., Smith C. R., Buschow A. W., Samadpour M. (2011). Polymerase chain reaction screening for Salmonella and Enterohemorrhagic Escherichia coli on beef products in processing establishments. *Foodborne Pathogens and Disease*.

[19] de Assis D. C., da Silva T. M., Brito R. F., da Silva L. C., Lima W. G., Brito J. C. (2020). Shiga toxin-producing *Escherichia coli* (STEC) in bovine meat and meat products over the last 15 years in Brazil: a systematic review and meta-analysis. *Meat Science*.

[20] KwagaTafida S. Y., fiBello J. K. P., Kabir J. (2014). Occurrence of Escherichia coli O157 in retailed-beef and related meat products in zaria, Nigeria. *Food and Nutrition Sciences*.

[21] Kiermeier A., Mellor G., Barlow R., Jenson I. (2011). Assumptions of acceptance sampling and the implications for lot contamination: *Escherichia coli* O157 in lots of Australian manufacturing beef. *Journal of Food Protection*.

[22] Samadpour M., Ongerth J. E., Liston J. (1994). Occurrence of Shiga-like toxin-producing Escherichia coli in retail fresh seafood, beef, lamb, pork, and poultry from grocery stores in Seattle, Washington. *Applied and Environmental Microbiology*.

[23] Brooks H. J. L., Mollison B. D., Bettelheim K. A., Matejka K., Paterson K. A., Ward V. K. (2001). Occurrence and virulence factors of non-O157 Shiga toxin-producing Escherichia coli in retail meat in Dunedin, New Zealand. *Letters in Applied Microbiology*.

[24] Gutema F. D., Rasschaert G., Agga G. E. (2021). Occurrence, molecular characteristics, and antimicrobial resistance of *Escherichia coli* O157 in cattle, beef, and humans in bishoftu town, Central Ethiopia. *Foodborne Pathogens and Disease*.

[25] Chinen I., Epszteyn S., Melamed C. L. (2009). Shiga toxin-producing *Escherichia coli* O157 in beef and chicken burgers, and chicken carcasses in Buenos Aires, Argentina. *International Journal of Food Microbiology*.

[26] Renter D. G., Sargeant J. M., Hungerford L. L. (2004). Distribution of Escherichia coli O157:H7 within and among cattle operations in pasture-based agricultural areas. *American Journal of Veterinary Research*.

[27] Elmonir W., Shalaan S., Tahoun A. (2021). Prevalence, antimicrobial resistance, and genotyping of Shiga toxin-producing *Escherichia coli* in foods of cattle origin, diarrheic cattle, and diarrheic humans in Egypt. *Gut Pathogens*.

[28] Noureldaim A. N., Abdul Mutalib S., Fufa Ido G., Wan Mohtar Y. (2019). Genetic characterization of shiga toxin-producing Escherichia coli strains isolated from imported beef meat in Malaysia using polymerase chain reaction analysis. *American Journal of Biological and Environmental Statistics*.

