# Supplementary figures and images for: Prevalence of Shiga Toxin-Producing Escherichia coli O157 and Non-O157 Serogroups Isolated from Fresh Raw Beef Meat Samples in an Industrial Slaughterhouse
Source: Int J Microbiol. 2021 Dec 15;2021:1978952. doi: 10.1155/2021/1978952 (PMC8695030; doi:10.1155/2021/1978952)

*stx*1


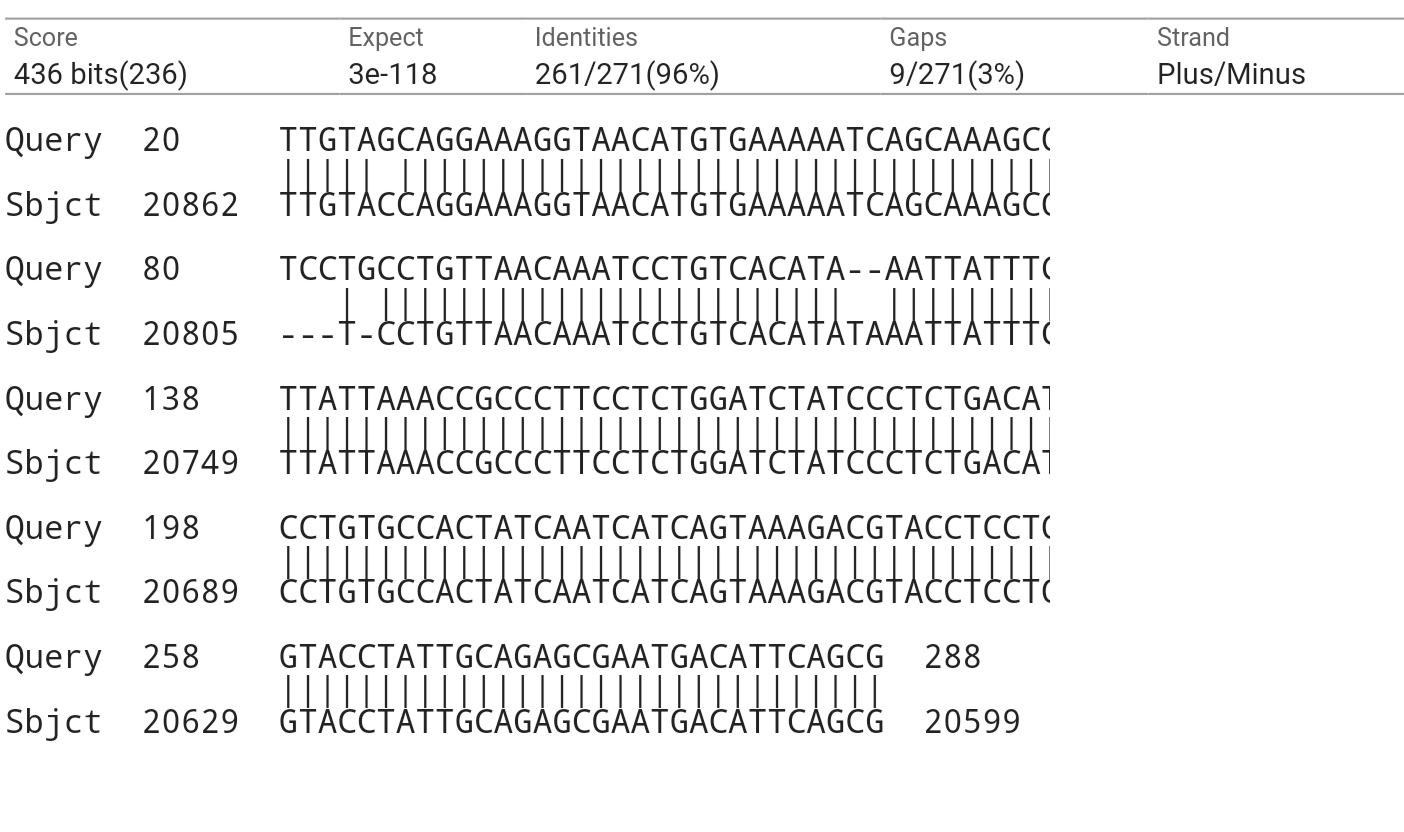


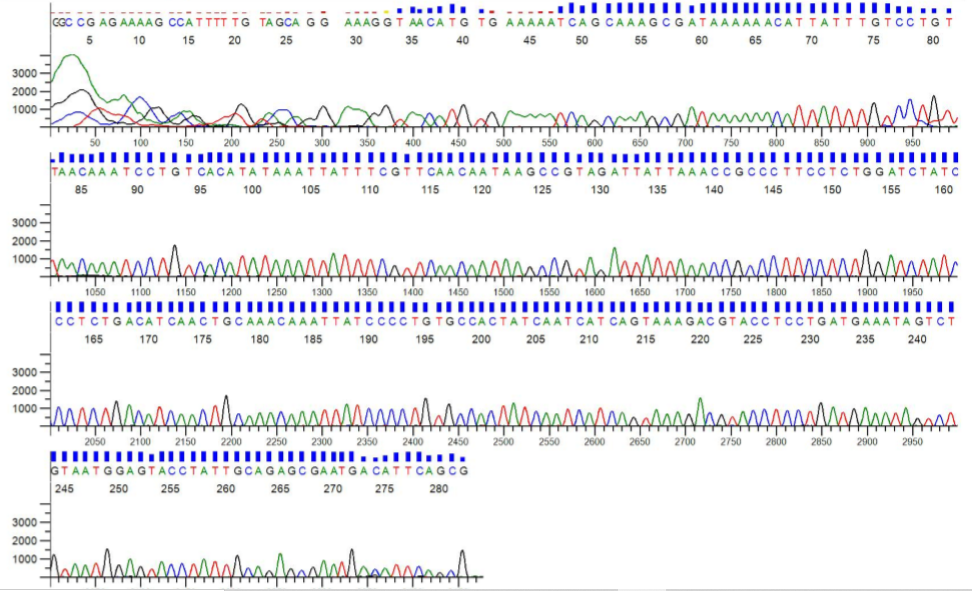


***stx2***


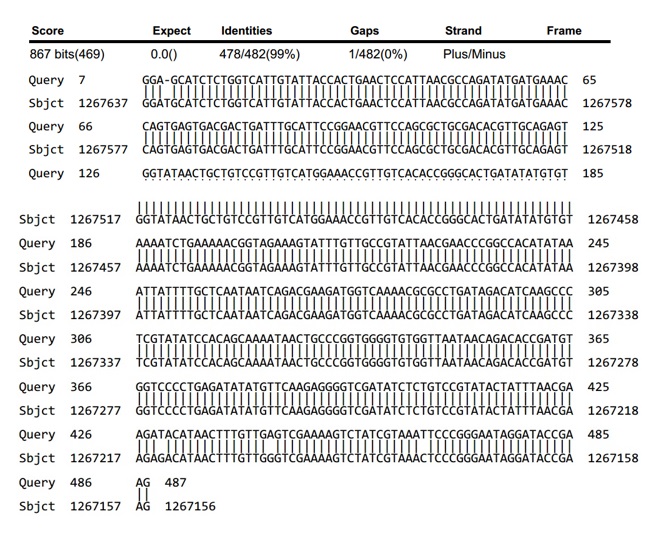


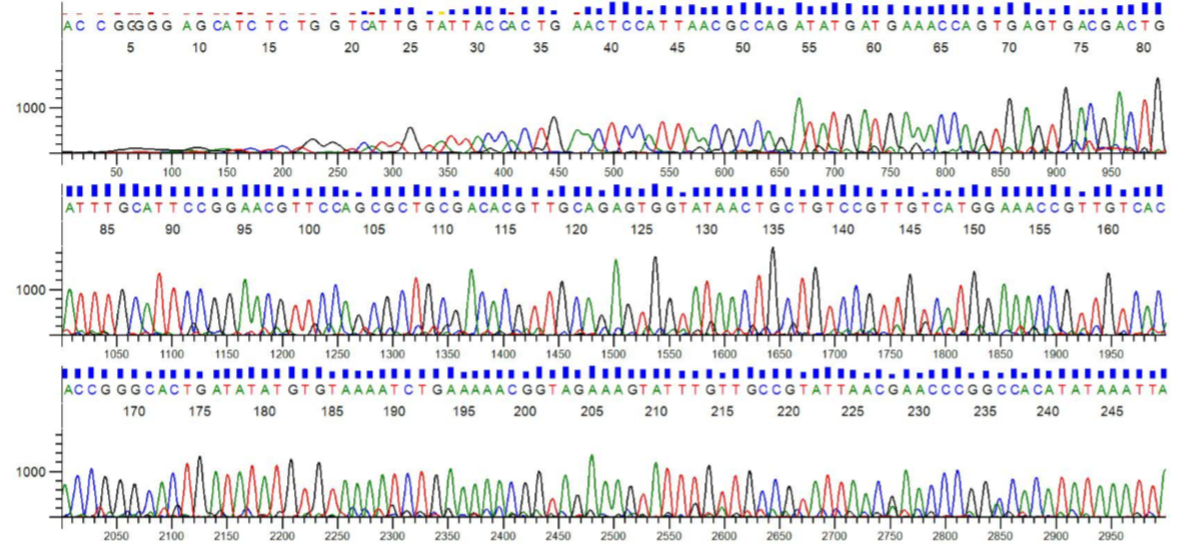


**
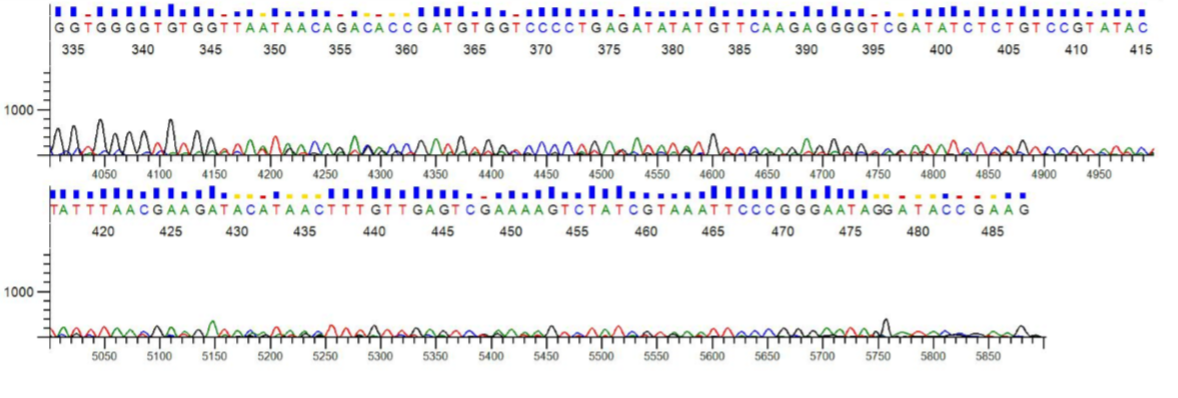
**

**O157 (*wzx*)**


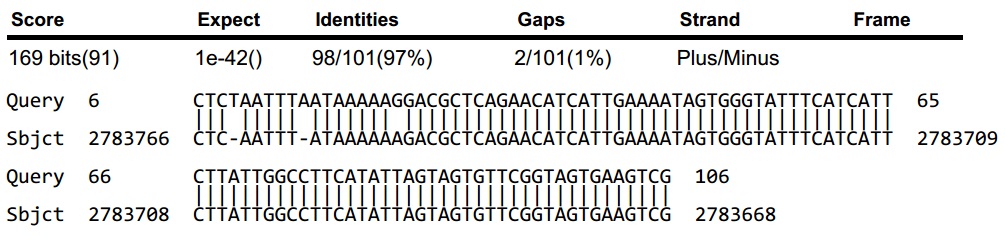


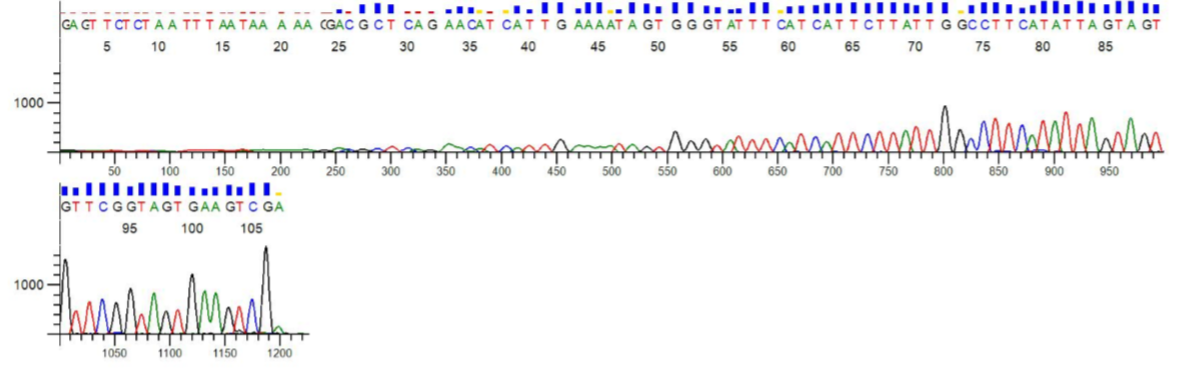

Supplement: Supplementary Materials — See the supplementary materials for the sequencing results of PCR products. [file 1978952.f1.docx]
